# Reproducibility of Standardized Uptake Values Including Volume Metrics Between TOF-PET-MR and TOF-PET-CT

**DOI:** 10.3389/fmed.2022.796085

**Published:** 2022-03-02

**Authors:** Aruki Tanaka, Tetsuro Sekine, Edwin E. G. W. ter Voert, Konstantinos G. Zeimpekis, Gaspar Delso, Felipe de Galiza Barbosa, Geoffrey Warnock, Shin-ichiro Kumita, Patrick Veit Haibach, Martin Huellner

**Affiliations:** ^1^Department of Radiology, Nippon Medical School Hospital, Tokyo, Japan; ^2^Department of Radiology, Nippon Medical School Musashi Kosugi Hospital, Kanagawa, Japan; ^3^Departments of Nuclear Medicine, University Hospital Zurich, Zurich, Switzerland; ^4^University of Zurich, Zurich, Switzerland; ^5^Department of Nuclear Medicine, Inselspital, Bern University Hospital, University of Bern, Bern, Switzerland; ^6^GE Healthcare, Waukesha, WI, United States; ^7^PMOD Technologies Ltd., Zurich, Switzerland; ^8^Toronto Joint Department Medical Imaging, University Health Network, Sinai Health System, Women's College Hospital, Toronto, ON, Canada; ^9^Department of Medical Imaging, University of Toronto, Toronto, ON, Canada

**Keywords:** PET/MR, PET/CT, reproducibility, metabolic tumor volume, FDG-F production 18, TOF (time-of-flight), SUV

## Abstract

**Purpose:**

To investigate the reproducibility of tracer uptake measurements, including volume metrics, such as metabolic tumor volume (MTV) and tumor lesion glycolysis (TLG) obtained by TOF-PET-CT and TOF-PET-MR.

**Materials and Methods:**

Eighty consecutive patients with different oncologic diagnoses underwent TOF-PET-CT (Discovery 690; GE Healthcare) and TOF-PET-MR (SIGNA PET-MR; GE Healthcare) on the same day with single dose−18F-FDG injection. The scan order, PET-CT following or followed by PET-MR, was randomly assigned. A spherical volume of interest (VOI) of 30 mm was placed on the liver in accordance with the PERCIST criteria. For liver, the maximum and mean standard uptake value for body weight (SUV) and lean body mass (SUL) were obtained. For tumor delineation, VOI with a threshold of 40 and 50% of SUVmax was used (VOI40 and VOI50). The SUVmax, SUVmean, SUVpeak, MTV and TLG were calculated. The measurements were compared between the two scanners.

**Results:**

In total, 80 tumor lesions from 35 patients were evaluated. There was no statistical difference observed in liver regions, whereas in tumor lesions, SUVmax, SUV mean, and SUVpeak of PET-MR were significantly underestimated (*p* < 0.001) in both VOI40 and VOI50. Among volume metrics, there was no statistical difference observed except TLG on VOI50 (*p* = 0.03). Correlation between PET-CT and PET-MR of each metrics were calculated. There was a moderate correlation of the liver SUV and SUL metrics (*r* = 0.63–0.78). In tumor lesions, SUVmax and SUVmean had a stronger correlation with underestimation in PET-MR on VOI 40 (SUVmax and SUVmean; *r* = 0.92 and 0.91 with slope = 0.71 and 0.72, respectively). In the evaluation of MTV and TLG, the stronger correlations were observed both on VOI40 (MTV and TLG; *r* = 0.75 and 0.92) and VOI50 (MTV and TLG; *r* = 0.88 and 0.95) between PET-CT and PET-MR.

**Conclusion:**

PET metrics on TOF-PET-MR showed a good correlation with that of TOF-PET-CT. SUVmax and SUVpeak of tumor lesions were underestimated by 16% on PET-MRI. MTV with % threshold can be regarded as identical volumetric markers for both TOF-PET-CT and TOF-PET-MR.

## Introduction

18F-fluorodeoxyglucose (FDG) positron emission tomography (PET) is used routinely in the diagnosis, staging, restaging, and treatment monitoring of various cancers (1). The maximum standardized uptake value (SUVmax) remains the main uptake measurement parameters of tumors, owing to its simplicity and high reproducibility. In order to achieve a more detailed assessment of tumor characteristics, recent studies have focused on demonstrating the prognostic value of positron emission tomography (PET)-based volumetric parameters, such as metabolic tumor volume (MTV) and total lesion glycolysis (TLG) (2–6). MTV is defined as the sum of the volume of voxels, and TLG is the product of the MTV and SUVmean. These indicators can be used for prognostication as they reflect the activity of glucose metabolism in the entire tumor compared to SUVmax which only reflects a single voxel value.

Following the success of the positron emission tomography and computed tomography (PET-CT) system, integrated PET and magnetic resonance (PET-MR) systems have been clinically introduced and the number of these scanners is gradually increasing worldwide (7–10). In clinical or research settings, several PET machines were used for identical clinical and research purposes. In such a situation, reproducibility among scanners remains an issue to be solved (11, 12). Especially on PET-MR, the error derived from the attenuation correction based on MRI (MRAC) impairs its reproducibility (13–23). Several studies have been conducted to assess the impact of this impairment. In these studies, the metrics of max, mean, or peak SUVs were mainly evaluated (24–26); however, the studies on the correlations of MTV and TLG between PET-CT and PET-MR are still very limited (27–31). Specifically, in the case of time-of-flight (TOF)-PET-MR instruments, no study has evaluated the volumetric parameters. In these machines, the effect of the MRAC error is reduced by TOF-reconstruction (32–34).

In this study, we aimed to determine the TOF-PET-CT and TOF-PET-MR reproducibility of FDG PET-SUV measurements, including volumetric metrics obtained by PET-CT and PET-MR examinations performed on the same day in oncologic patients.

## Materials and Methods

### Ethical Statement

Patients were enrolled in this retrospective study as part of a larger prospective study (NCT02316431). All patients provided written informed consent prior to their inclusion in the study.

### Study Subjects

Eighty consecutive patients with different oncologic disease underwent 18F-FDG PET-CT and 18F-FDG PET-MR. All examinations were performed on the same day with a single injection of FDG. The inclusion criteria called for patients with visible tumors both on PET-CT and PET-MR. The scan interval between both examinations was <70 min. A sufficient tracer activity was maintained to generate PET images on PET-MR equivalent to a 70% dose of PET-CT (35).

### Image Acquisition

PET-CT acquisition followed a standard protocol for clinical oncologic imaging on a TOF-PET-CT scanner (Discovery 690; GE Healthcare, Waukesha, Wisconsin, USA) (12). The whole-body PET data were acquired in 3D TOF mode with a scan duration of 2 min per bed position, an axial FOV of 153 mm, and 23% overlap of bed positions—resulting in a total PET acquisition time of 16–20 min. Standard CT was acquired for diagnostic purposes and CT attenuation correction (CTAC).

For the PET-MR imaging examination, we used a simultaneous TOF-PET-MR system, which comprised a 3.0-T whole-body MR imaging system and SiPM PET detectors (SIGNA PET-MR; GE Healthcare, Waukesha, Wisconsin, USA). Whole-body list-mode PET data were acquired in 3D TOF mode with a scan duration of 2–4 min per bed position. The scan time per bed position depended on the imaging protocol selected according to clinical indication. An axial FOV of 250 mm and a 24% overlap of bed positions were used, resulting in a total PET acquisition time of 12–24 min. During PET-MR imaging, a 3D liver acquisition with volume acquisition (LAVA Flex) T1-weighted pulse sequence (repetition time—~4 ms; echo time−2.23 ms; flip angle−5°; section thickness−5.2 mm with 2.6 mm overlap; 120 sections; pixel size−1.95 × 1.95 mm^2^, partial Fourier−70.3%; and acquisition time−18 s per bed position) was acquired for MRAC (33, 36). Additionally, different anatomic MR pulse sequences were also used for diagnostic imaging.

### Imaging Reconstruction

The reconstruction parameters of PET images on PET-CT and PET-MR were selected to be as similar as possible. The detailed parameters were described elsewhere (35). In PET-CT, a fully 3D OSEM iterative reconstruction, including PSF compensation with three iterations and 18 subsets and a 256 × 256 image grid (2.73 × 2.73 × 3.27-mm voxels), was used. In PET-MR imaging, OSEM, including PSF compensation with three iterations and 16 subsets, and a 256 × 256 image grid (2.34 × 2.34 × 2.78-mm voxels), was used for reconstruction of the PET images. In both systems, transaxial post-reconstruction Gaussian filter 4 mm, axial filter 1:4:1, normalization, random, scatter, dead-time and decay correction were applied. The parameters of PET-CT had been fixed as clinical scan. The minor difference of parameters between both scanners were due to the restriction by the vendor. We could not choose the parameter freely but one out of several options. Both PET image datasets were generated by TOF calculation. CTAC was used to generate PET on PET-CT and MRAC for PET on PET-MR. To compensate for the difference in sensitivity between the two scanners derived from SiPM detectors on PET-MR, we retrospectively un-listed the list mode PET data on PET-MR and generated 70% simulated-dose PET-MR images comparable to PET-CT (35).

### Imaging Analysis

We extracted normal liver regions and oncologic lesions for further evaluation. As normal liver regions, the liver mean SUV (SUVmean) normalized to lean body mass (SULmean) was measured. These values have been proposed as a quality control measure for FDG PET-CT in solid tumors (PERCIST) 2.0 (12). A VOI with a diameter of 3 cm was manually drawn on the right lobe of the liver to analyze the concordance between PET-CT and PET-MR.

As target lesions, a maximum of three tumors were extracted per each of the four body parts (head and neck, chest, upper abdomen, and pelvis) by two independent board-certified radiologists (T.S and B.F). Tumors larger than 25 mL were excluded to maintain stability in the statistical analysis. We also excluded target lesions that could not reliably be delineated from physiological uptake (such as the heart, kidney, and bladder).

The volume-of-interest (VOI) was defined by manually drawing polygonal VOIs to enclose the entire tumor with sufficient margins on every slice where the target tumor was seen. Physiological uptake was carefully avoided. In this study, we used the fixed 40 and 50% to SUVmax threshold method (VOI40 and VOI50, respectively), which is a procedure for defining the area of the tumor as a region with a higher SUV than a certain percentage of the SUVmax within the tumor (12).

We used PMOD (version 4.0; PMOD Inc., Zurich, Switzerland) for VOI segmentation and calculation of SUVmax, SUVpeak, SUVmean, MTV, and TLG. VOIs below 1 mL were excluded for the static of peak (37).

### Statistical Analysis

We performed the Kolmogorov–Smirnov test for the continuous variables. The values of liver SUV and SUL were normally distributed and listed as mean ± standard deviation. The values of tumor SUV were not normally distributed and listed as median and interquartile range (IQR). To clarify the difference in PET metrics between the two scanners, we performed a paired *t*-test for normally distributed variables and Wilcoxon signed-rank test for not normally distributed variables, respectively. In order to prove the correlation between the two scanners, Pearson's test was performed for these metrics as well. To visualize the deviation of the difference, Bland–Altman plots with limits of agreement were generated (38). Statistical significance was set at *p* < 0.05. Statistical analyses were performed using SPSS Statistics, version 19.0.0 (IBM, Armonk, NY, USA).

## Results

**Figure 1:**
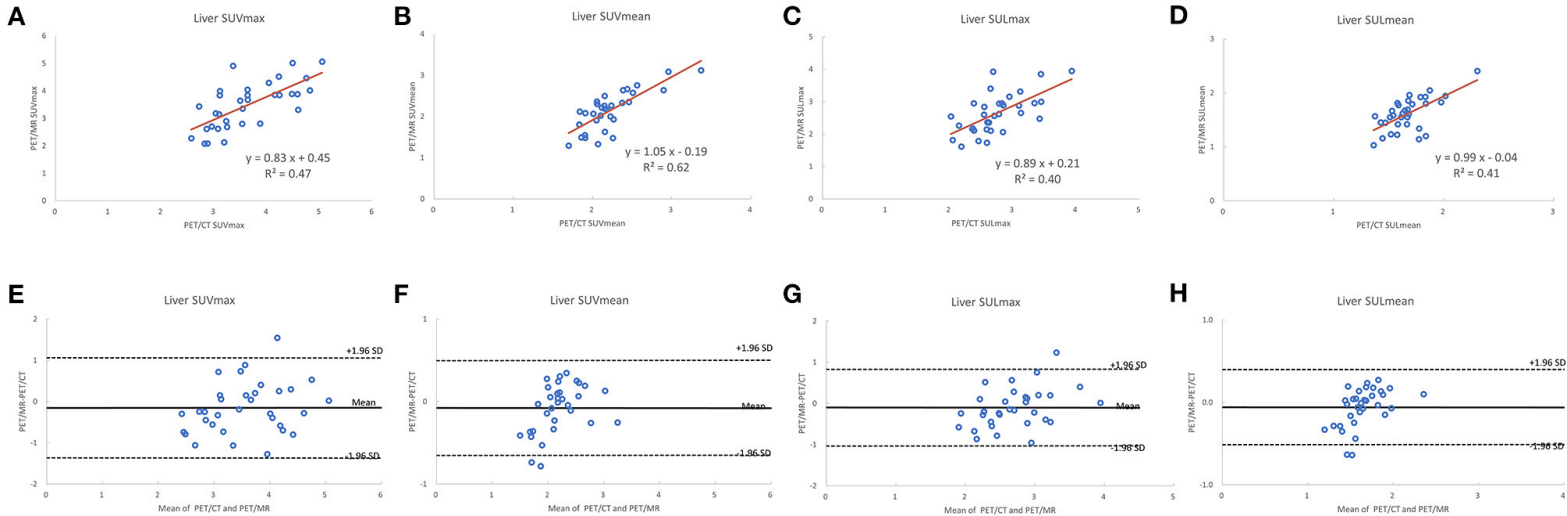
Scatter diagram with regression line and Bland-Altman plots of PET-CT and PET-MR for SUVmax and SUVmean corrected to body weight **(A,B,E,F)** and lean body mass **(C,D,G,H)**.

**Figure 2:**
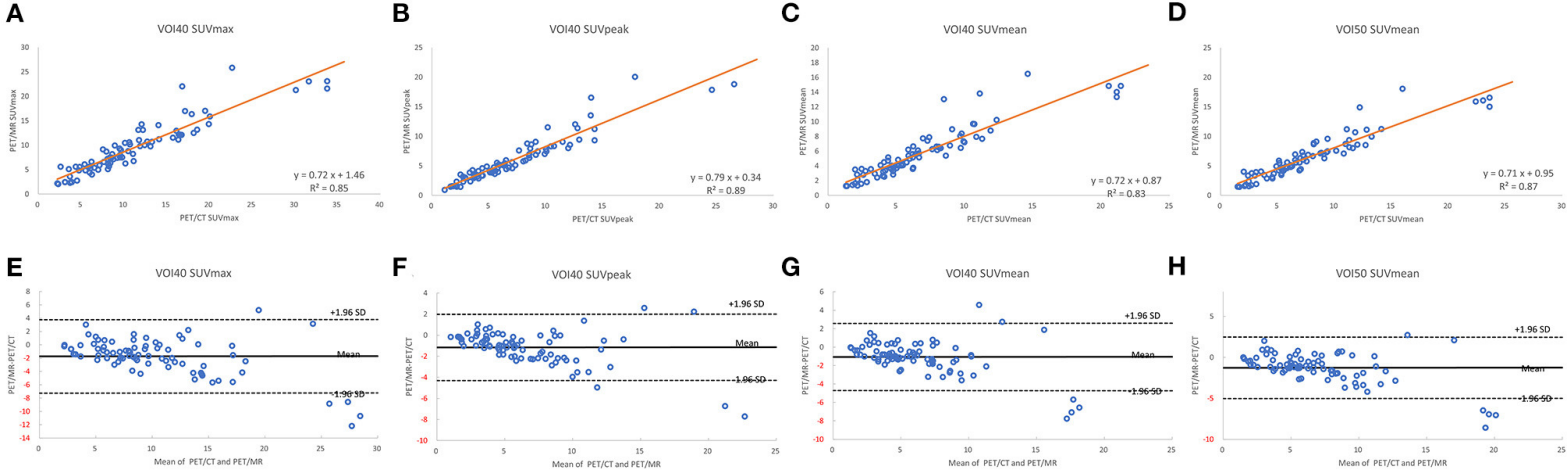
Scatter diagram with regression line and Bland-Altman plots of PET-CT and PET-MR for SUVmax **(A,E)**, SUVpeak **(B,F)**, and SUVmean **(C,G)** of VOI40 and SUVmean of VOI50 **(D,H)**.

**Figure 3:**
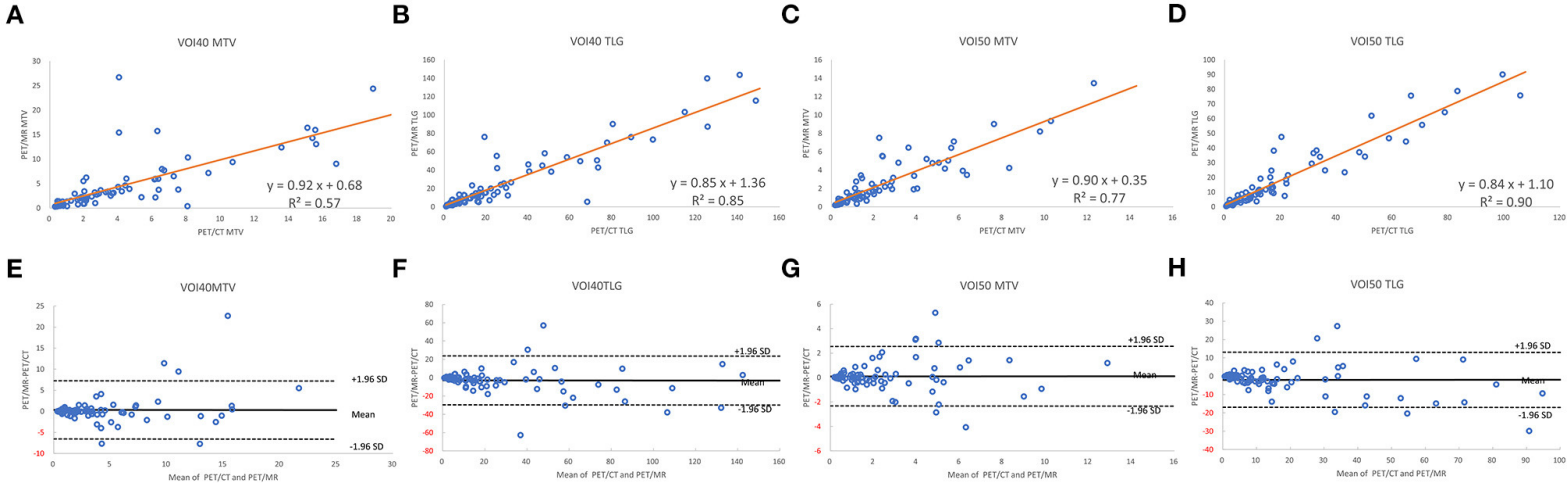
Scatter diagram with regression line and Bland-Altman plots of PET-CT and PET-MR for MTV and TLG of VOI40 **(A,B,E,F)** and VOI50 **(C,D,G,H)**.

**Figure 4:**
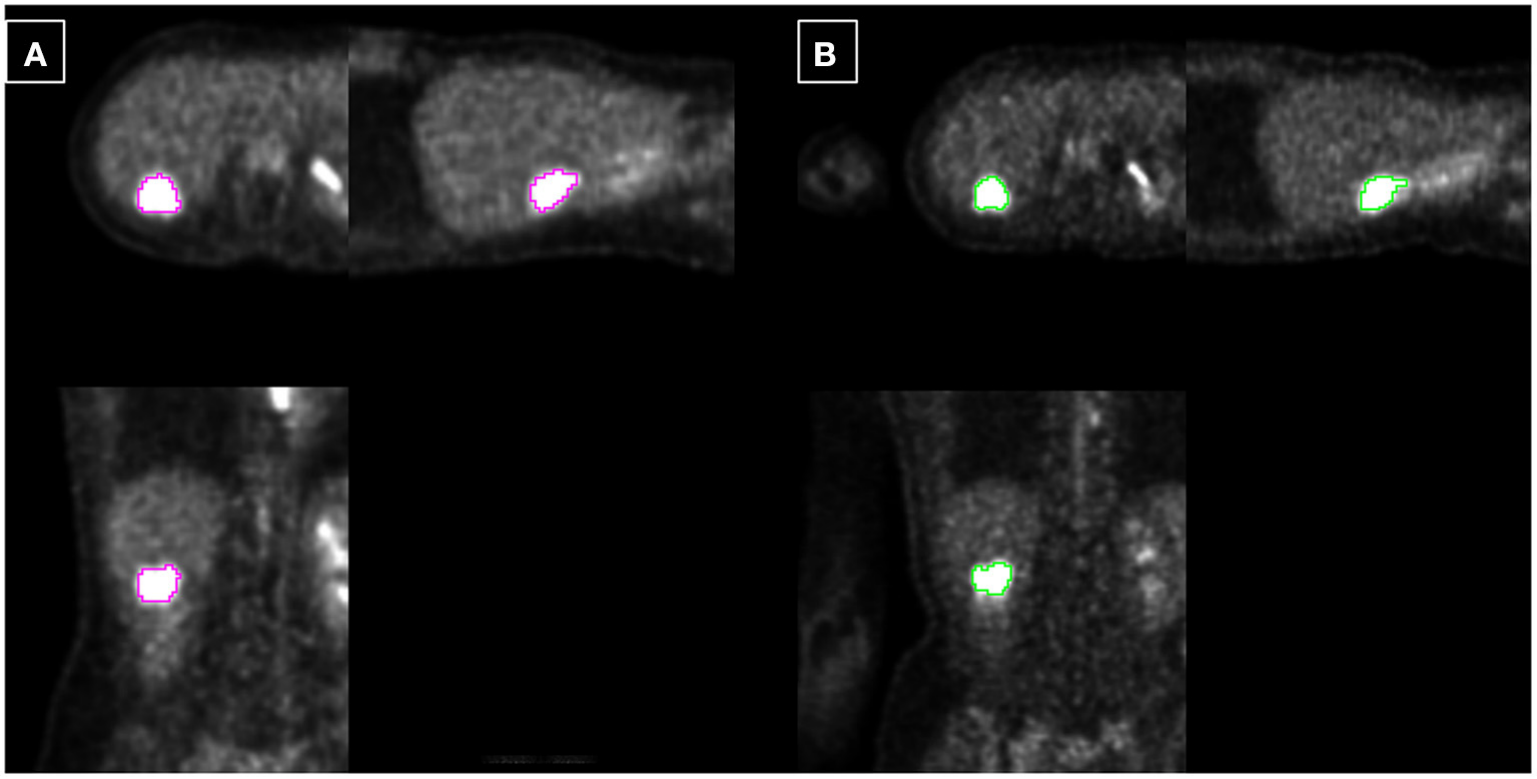
Example 1: 40-year-old female with liver metastasis of breast cancer. Each PET-CT **(A)** and PET-MR **(B)** was acquired 72 and 115 min after injection. The tumor is delineated based on fixed 40% threshold to SUVmax.

**Figure 5:**
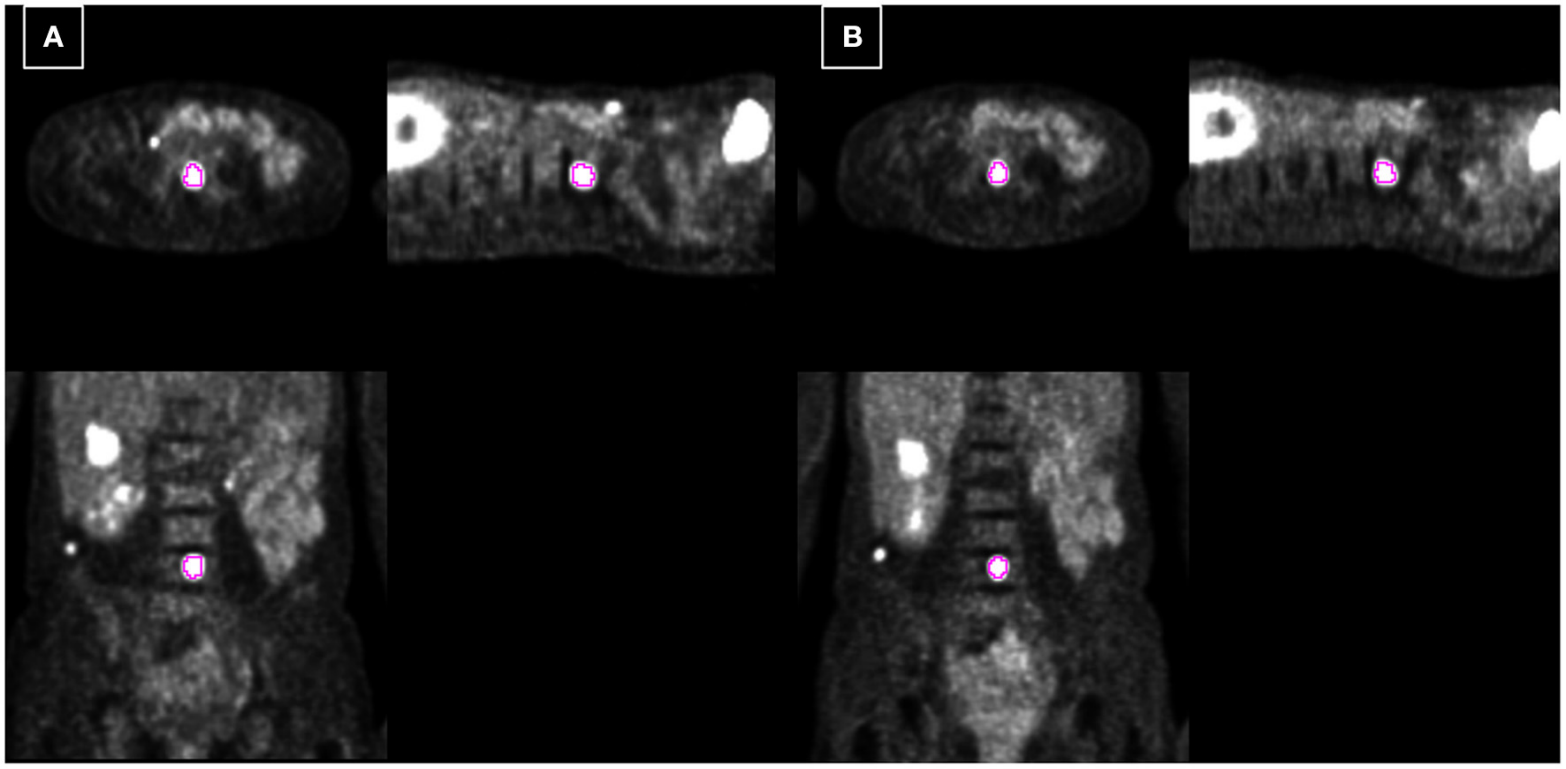
Example 2: 54-year-old female with spine metastasis of melanoma. Each PET-CT **(A)** and PET-MR **(B)** was acquired 73 and 48 min after injection. The tumor is delineated based on fixed 40% threshold to SUVmax.

A total of 35 patients with 80 tumor lesions were included. The detailed information is given in Table 1. The mean and standard deviation of each metric on both PET-CT and PET-MR is given in Table 2. There was no statistical difference observed in the liver regions, whereas the tumor SUVmax, and SUVpeak of PET-MR were significantly underestimated (*p* < 0.001). Among volume metrics consisting of MTV and TLG, there was no statistical difference observed except for the TLG of VOI50 (*p* = 0.03). The correlation analysis between PET-CT and PET-MR is given in Table 3. The correlation of the liver SUVs and SULs was moderate (*r* = 0.63–0.78) (Figures 1A–D). In tumor lesions, SUVmax, SUVmean, and SUVpeak were strongly correlated with an underestimation on PET-MR (*r* = 0.92, 0.91, and 0.95, respectively; slope = 0.71, 0.72, and 0.79, respectively) (Figures 2A–D). For MTV and TLG, high correlations were observed with slightly better results on VOI50 compared to VOI40 (0.88 and 0.95 vs. 0.75 and 0.92, respectively) (Figures 3A–D). The results of the Bland-Altman analysis are presented in Table 4. Mean differences of all measurements were negative except MTV in VOI40 and VOI50 (Figures 1E–H, 2E–H, 3E–H). The representative cases are shown in Figures 4, 5.

**Table 1:** Demographic and clinical data.

Age in years, mean ± SD (range)	63.1 ± 10.7 (40–84)
Body height in meters, mean ± SD (range)	1.7 ± 0.1 (1.49–1.9)
Body weight in kilograms, mean ± SD (range)	71.7 ± 15.2 (44–110)
BMI, mean ± SD (range)	24.5 ± 4.1 (17.4–32.7)
Injected dose in MBq/kg, mean ± SD (range)	3.20 ± 0.30 (2.84–4.07)
Gender (*n*)	
Male	22
Female	13
Clinical indication (*n*)	
Head and neck cancer	9
Lung cancer	8
Pancreatic cancer	3
Breast cancer	2
Esophageal cancer	2
Rectal cancer	2
Cancer of unknown primary	2
Malignant lymphoma	2
Colon cancer	2
Intrahepatic cholangiocarcinoma	1
Multiple myeloma	1
Malignant melanoma	1
PET-MR images acquired after injection in minutes, mean ± SD (range)	75 ± 12 (46–104)
PET-CT images acquired after injection in minutes, mean ± SD (range)	63 ± 26 (37–144)
Scan interval (PET-MR minus PET-CT) (min), mean ± SD (range)	−12 ± 32 (−54–68)

**Table 2:** Details of SUV measurements in liver and tumor lesions.

			**PET-CT** **mean ±SD**	**PET-MR** **mean ±SD**	***P*** **(paired *t*-test)**
Liver		SUVmax	3.63 ± 0.70	3.48 ± 0.84	0.17
		SUVmean	2.21 ± 0.35	2.13 ± 0.47	0.14
		SULmax	2.74 ± 0.44	2.64 ± 0.61	0.22
		SULmean	1.67 ± 0.20	1.61 ± 0.30	0.16
			**PET-CT** **median** **±IQR**	**PET-MR** **median** **±IQR**	**(Wilcoxon** **signed-rank test)**
Tumor lesion	VOI40	SUVmax	9.54 (6.32–14.09)	8.23 (5.62–12.18)	<0.001
		SUVpeak	6.11 (3.80–9.24)	4.98 (3.44–7.92)	<0.001
		SUVmean	5.92 (4.09–8.57)	5.07 (3.52–7.31)	<0.001
		MTV	2.30 (0.78–6.15)	2.34 (0.92–5.97)	0.850
		TLG	15.35 (4.07–31.38)	12.42 (4.07–38.60)	0.002
		SUVmean	6.57 (4.94–9.38)	5.60 (4.04–8.13)	<0.001
	VOI50	MTV	1.42 (0.45–2.87)	1.50 (0.59–3.43)	0.694
		TLG	9.54 (2.80–21.75)	8.10 (2.67–23.02)	0.002

**Table 3:** The result of linear regression analysis where x-axis is PET/MR measurements and y-axis is PET/CT measurements.

			**Slope**	**Intercept**	***r***
Liver		SUVmax	0.83	0.45	0.69
		SUVmean	1.05	−0.19	0.78
		SULmax	0.89	0.21	0.63
		SULmean	0.99	−0.04	0.64
Tumor lesion	VOI40	SUV max	0.71	1.48	0.92
		SUVpeak	0.80	0.35	0.94
		SUVmean	0.72	0.87	0.91
		MTV	0.92	0.68	0.75
		TLG	0.85	1.24	0.92
	VOI50	SUVmean	0.71	0.95	0.93
		MTV	0.90	0.35	0.88
		TLG	0.84	0.98	0.95

**Table 4:** The result of Bland-Altman analysis of SUV measurements between PET/CT and PET/MR.

			**Mean difference (PET-MR – PET-CT)**	**Limits of agreement**	
				**Lower**	**Upper**
Liver		SUVmax	−0.15 ± 0.62	−1.37	1.06
		SUVmean	−0.08 ± 029	−0.65	0.50
		SULmax	−0.10 ± 048	−1.03	0.83
		SULmean	−0.06 ± 023	−0.51	0.40
Tumor lesion	VOI40	SUVmax	−1.71 ± 2.83	−7.22	3.80
		SUVpeak	−1.25 ± 1.76	−4.69	2.19
		SUVmean	−1.06 ± 1.87	−4.71	2.59
		MTV	0.35 ± 3.55	−6.57	7.27
		TLG	−2.94 ± 13.75	−29.76	23.88
	VOI50	SUVmean	−1.26 ± 1.92	−5.00	2.48
		MTV	0.11 ± 1.25	−2.33	2.55
		TLG	−1.86 ± 7.70	−16.88	13.15

## Discussion

This study demonstrated that SUV measurements correlate well between PET-MR and PET-CT. SUVmax and SUVmean of the reference area, liver regions, were not under- or overestimated on PET-MR. However, the SUVmax, SUVpeak, and SUVmean of tumor lesions were underestimated by ~16%. Fortunately, MTV was maintained between both scanners.

There are notable strengths to the current study. First, we evaluated the reproducibility of volume metrics between PET-CT and PET-MR, which was evaluated only by the sole previous study where non-TOF-PET-MR machines were used (27). TOF-reconstruction clearly compensates for the error from the MRAC (32, 33). For this reason, their results cannot be transferred to a TOF-PET-MR machine, but separate analysis is required. Second, the scan order, PET-MR following or followed by PET-CT, was randomly in our study. Delayed uptake is a critical issue when comparing the reproducibility (39). Third, we generated PET images on PET-MR equivalent to a 70% dose of PET-CT. The higher sensitivity of SiPM detectors or novel reconstruction techniques can influence PET metrics (40, 41). For a fair comparison regardless of detector sensitivity, we developed the current study design.

Although the extent of the difference was within the limit of repeatability (i.e., 25%) (42), a significant underestimation of several parameters was observed on PET-MR. The underestimation of SUVmax, mean, and peak is expected to be derived from two factors. One is that incomplete MRAC based on 4-compartment models which consist of air, lung, fat, and soft tissue. Neglecting bone tissues causes an underestimation of SUV uptake more than 10% (13, 43). The incorrect estimation of bone tissue AC correction factors affect mostly PET signal quantification or other regions proximity to bone and this effect becomes negligible for regions at a larger distance from bone tissues. This might explain why the SUV metrics of the liver were not statistically different between the two scanners. Around the liver, there is no solid bone except for the thin rib bone, which results in maintaining the accuracy of attenuation correction. It may cause secondary critical problems in the calculation of the tumor/liver ratio or tumor delineation from the uptake of the liver. To improve upon the insufficiency of MRAC, model-based bone imposition (44), bone estimation using ZTE/UTE MRI (45, 46), and deep-learning methods (45) have been proposed. In addition, dual-tracer approach where one of the tracers is that of interest and the other may be 18F Sodium Fluoride, NaF, from which the bone can be segmented (20). However, other than the model-based methods, an implementation into clinical scans has yet to occur. Another factor is that the reconstruction parameters were set to be as similar as possible, but were not identical for both scanners. The reason is that scanner software restricted users tune parameters freely (e.g., voxel resolution).

Compared to the previous MTV-reproducibility research by Groshar et al. (27), the bias of MTV in the current study was smaller (3.0 vs. 27.3%); this may be because we had a smaller scan interval (−12 ± 32 min in the current study vs. 53 ± 17 min in the previous study). In contrast, the 95% limits of agreement had a larger range (−96.5 and 102.5 vs. −41.7 to 96.2%). This might be owing to our inclusion criteria. Whereas the previous study included extremely large tumors (> 150 mL), we chose to exclude tumors larger than 25 mL to maintain statistical stability. Four previous studies evaluated the reproducibility between TOF-PET-CT and TOF-PET-MR using the same commercial scanner, GE SIGNA-PET-MR; however, none of these studies performed a truly “fair” comparison between the two scanners in terms of a fixed scan order (i.e., PET-CT following or followed by PET-MR) with a long scan interval (e.g., more than 80 min) (25, 47–49). Our study represents the first report using a random sequence of PET-CT and PET-MR acquisition, with a comparably short scan interval.

Based on our results, the SUVmax or SUVpeak metrics should be carefully considered when both PET-CT and PET-MR machines are used in follow-up or multi-center studies (50). Unlike SUVmax, SUVmean, or SUVpeak, there was no statistical difference in MTV between the two scanners. One can speculate that the tumor volume was determined by the ratio of maximum uptake and the SUV on the edge of the delineation. Therefore, if the tumor is uniformly underestimated by MRAC, the MTV does not change.

Our study has a few limitations. First, we used the 70%dose un-list PET-MR, hence evaluated data may differ from clinical data. However, the purpose of this study was to perform a fair comparison of PET-CT and PET-MR, regardless of detector sensitivity or advanced reconstruction techniques. Second, both the scan time and scan interval of PET-CT and PET-MR were not uniform. For example, some PET-CT scanned after 144 min, which was quite over from recommended protocol for PET-CT (50–70 min according to PERCIST) (51). In the delayed acquisition, the SUV-increase in tumor and SUV-decrease in benign lesion were expected, as FDG accumulation of tumor has unique characteristic, Warburg Effect, which is different from the characteristic of physiological uptake (52–56). One must consider this limitation when interpreting the current result of each liver and tumor, respectively. This limitation represents an inherent problem for a reproducibility study because repeated injection of tracer is ethically hard to justify. The same-day repeatability ruled out additional sources of quantitative error deriving from patient habitus or the progression/regression of tumors between the two scans. Third, there might be some room to adjust the parameter of PET-MR to PET-CT, although we chose both parameters as same as possible. For further implementation of the current result into clinical research such as multi-center study, the phantom-validation applying multiple reconstruction parameters would be required. In such a case, to achieve harmonization, an automatic and secondary reconstruction of the PET-MR images which match PET-CT images would be practical (50).

## Conclusion

PET metrics from TOF-PET-MR had good correlation to those from TOF-PET-CT. SUVmax and SUVpeak of tumor lesions were underestimated by 16% on PET-MR. Careful consideration should be paid to the difference of the extent of underestimation between reference tissue (liver) and target tissue (tumor) when semi-quantitative parameters are measured. MTV with a % threshold can be utilized as the identical volumetric markers both on TOF-PET-CT and TOF-PET-MR.

## Data Availability Statement

The raw data supporting the conclusions of this article will be made available by the authors, without undue reservation.

## Ethics Statement

The studies involving human participants were reviewed and approved by the Ethics Committee of University Hospital Zurich. The patients/participants provided their written informed consent to participate in this study.

## Author Contributions

TS and AT conceived the presented idea, developed the theory, performed the computations, and verified the analytical methods. MH and PV encouraged TS to investigate using this data and supervised the findings of this work. All authors discussed the results and contributed to the final manuscript.

## Conflict of Interest

GD is an employee of GE Healthcare. GW is an employee of Pmod Inc. The remaining authors declare that the research was conducted in the absence of any commercial or financial relationships that could be construed as a potential conflict of interest.

## Publisher's Note

All claims expressed in this article are solely those of the authors and do not necessarily represent those of their affiliated organizations, or those of the publisher, the editors and the reviewers. Any product that may be evaluated in this article, or claim that may be made by its manufacturer, is not guaranteed or endorsed by the publisher.
